# Repellent and Insecticidal Efficacy of Four Water‐Soluble Formulations of Essential Oils Against *Aedes aegypti*

**DOI:** 10.1155/jotm/2881209

**Published:** 2026-02-16

**Authors:** Heber Silva-Díaz, Emma Vanesa Arriaga-Deza, Angie Vilma Serrato-Monja, Sebastian Iglesias-Osores, Lizzie Karen Becerra-Gutiérrez

**Affiliations:** ^1^ Faculty of Human Medicine, University of San Martín de Porres, Chiclayo, Peru, usmp.edu.pe; ^2^ Research Department, Lambayeque Regional Hospital, Chiclayo, Peru; ^3^ Faculty of Biological Sciences, Pedro Ruiz Gallo National University, Lambayeque, Peru; ^4^ Department of Entomology, Faculty of Agronomy, La Molina National Agrarian University, Lima, Peru, lamolina.edu.pe; ^5^ Department of Research, Registration and Development, Clenvi SAC, Lima, Peru

**Keywords:** *Aedes aegypti*, DEET, dengue (MeSH-NLM), insect repellents, oils, volatile

## Abstract

**Introduction:**

Arboviral diseases transmitted by the *Aedes aegypti* mosquito cause high morbidity and mortality worldwide.

**Objective:**

To evaluate the repellent and insecticidal efficacy of water‐soluble formulations of essential oils impregnated into mosquito nets against the adult stage of *A. aegypti*.

**Methodology:**

A randomized laboratory bioassay was conducted using a controlled factorial design to evaluate four essential oil formulations (*Eucalyptus globulus, Cymbopogon citratus, Origanum vulgare, and Mentha piperita*) at concentrations of 10, 100, and 250 mg/mL. Each experimental group consisted of 60 laboratory‐reared adult female *A. aegypti* specimens. The repellent effect, protection time, and insecticidal effect were evaluated by exposing the insects to a sedated animal bait (*Rattus norvegicus albinus*) covered with a mosquito net impregnated with the formulations of essential oils and DEET, as a positive control.

**Results:**

The highest efficacy was seen in *C. citratus, E. globulus*, and *O. vulgare* at 250 mg/mL, with 93.3%–100.0% repellent effect and 180 min of protection time, similar to that obtained by 100 mg/mL DEET. The highest insecticidal effect was observed in *C. citratus* (26.7%) at a concentration of 250 mg/mL, similar to that observed in 100 mg/mL DEET. The repellent effect and protection time varied for each concentration tested, except for the insecticide, in which concentrations of 10 and 100 mg/mL had a similar effect (0%–6%).

**Conclusions:**

The repellency of water‐soluble formulations of essential oils of *C. citratus*, *E. globulus*, and *O. vulgare* at 250 mg/mL, compared to 100 mg/mL DEET, represents a possible and complementary alternative for mosquito control.

## 1. Introduction

In recent years, dengue cases have increased worldwide, which, together with Zika and chikungunya, have created a complex and challenging epidemiological situation for public health systems, mainly in tropical and subtropical countries [1]. According to the World Health Organization (WHO), dengue cases increased from 505,430 in 2000 to 14.6 million in 2024 [2].

Indeed, according to the WHO, dengue is endemic in more than 100 countries in the regions of Africa, the Americas, Southeast Asia, the Eastern Mediterranean, and the Western Pacific [2]. In the region of the Americas, as of epidemiological week 27 of 2025, more than 3,449,053 suspected cases of dengue have been reported [3]. In Peru, dengue is classified as an epidemic, with the regions of Piura, Loreto, Lambayeque, Ica, La Libertad, and Lima being the most affected. According to reports from the Ministry of Health (MINSA), 33,759 cumulative cases have been reported so far in 2025 [4].

Moreover, the increasing resistance to inorganic insecticides, coupled with their toxicity to ecosystems and humans, has limited their use in public health to control the population of *Aedes aegypti*, the mosquito that transmits dengue and other arboviral diseases [5].

In this context, avoiding *A. aegypti* mosquito bites has become an important strategy for the prevention and control of the disease. Therefore, the use of insect repellents has been successfully extended, primarily synthetic repellents such as diethyltoluamide (DEET), picaridin, *p*‐menthano‐3,8‐diol (PMD), diethyl phenyl acetamide (DEPA), and ethyl butylacetylaminopropionate (IR3535) [6]. However, their toxic effects on the environment and humans have not yet been fully described, particularly for DEET, one of the most widely used repellents in Peru [7].

Therefore, the search for new chemical principles with repellent potential and low toxicity is a current need. In this regard, certain natural compounds of plant origin, such as essential oils, have emerged as alternatives to synthetic options [7]. Indeed, a previous local study showed the repellent potential of the essential oils from several plant species against *A. aegypti*, with *Eucalyptus globulus*, *Cymbopogon citratus,* and *Mentha piperita* standing out for their extraction yield and efficacy [8]. However, their behavior as water‐soluble formulations impregnated into mosquito nets is still unknown.

The purpose of this study was to evaluate the repellent and insecticidal efficacy of formulations of essential oils with repellent potential impregnated into mosquito nets against the adult stage of *A. aegypti*.

## 2. Materials and Methods

### 2.1. Experimental Type and Design

Laboratory bioassay with a factorial (4Fa x 3Fb), controlled, randomized, and single‐blinded arrangement was conducted in triplicate. A sample size of 2520 adult female *A. aegypti* specimens ([four formulations of essential oils x three concentrations x three replicates x 60 specimens per experimental group] + [two controls x three replicates x 60 specimens per experimental group]) was used (see Table 1).

**TABLE 1:** Laboratory bioassay with factorial design.

Group	Fa: treatment	Fb: concentrations	Outcome
Formulations of essential oils	*Eucalyptus globulus* *Cymbopogon citratus* *Mentha piperita* *Origanum vulgare*	10 mg/mL 100 mg/mL 250 mg/mL	Repellent effect (%)

Positive control	DEET (synthetic repellent)	100 mg/mL	Protection time (minutes)

Negative control	Distilled water and solvent solution in a 1:1 ratio	0 mg/mL	Insecticidal effect (%)

*Note:* DEET: N,N‐diethyl‐*meta*‐toluamide. Experimental model: *Aedes aegypti.* Bait*: Rattus norvegicus albinus.* Solvent solution: ethyl alcohol and 8% polysorbate.

**FIGURE 1:**
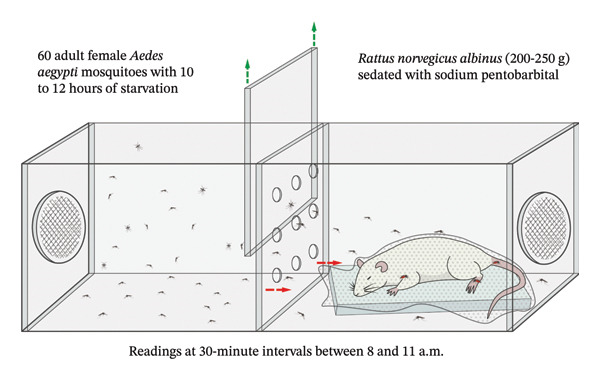
Design of the laboratory bioassay for collecting data on repellency, protection time, and insecticidal effect.

The afternoon before, 60 specimens were randomly distributed from the rearing cages to each of the two‐compartment cages where the laboratory bioassay was conducted. The two‐compartment cages were then randomly assigned by an operator who was not involved in the laboratory bioassay, using labels with alphabetical codes for each treatment. The replicates were performed on different days and in cages with fresh specimens of *A. aegypti* (see Figure 1).

### 2.2. Biological Material

The four essential oils were *M. piperita, E. globulus, O. vulgare, and C. citratus*, selected based on a local study, taking into account their extractive yield, repellency, and protection time [8]. The plants were collected in the Reque District, Lambayeque, Peru, and kept at the Herbarium of the School of Biological Sciences of the Universidad Nacional Pedro Ruiz Gallo, where they were botanically characterized (Table 2). Subsequently, they were transferred to the laboratory of the School of Chemical Engineering and Food Industries of the same university, where the essential oils were extracted by the steam stripping technique. The temperature was set at 80°C for approximately 60 min, except for *E. globulus*, which required 90 min. The yield was 2.51%, 1.97%, 1.25%, and 0.75% for *E. globulus*, *O. vulgare*, *M. piperita*, and *C. citratus*, respectively. DEET was purchased commercially from analytical standard (PESTANAL).

**TABLE 2:** Botanical and collection site characteristics of biological material.

Scientific name (common name)	Family	Plant type	Local use	Collection location (DMS)
*Cymbopogon citratus* (hierba luisa)	Poaceae	Grass	Food and medicine	Reque (−6.839215, −79.786216)
*Mentha piperita* L. (menta)	Lamiaceae	Grass	Medicinal and food	Reque (−6.839215, −79.786216)
*Origanum vulgare* (orégano)	Lamiacea*e*	Grass	Medicinal and food	Reque (−6.839215, −79.786216)
*Eucalyptus globulus* Labillardiere (eucalipto)	Myrtaceae	Tree	Ornamental, wood, and medicinal	Monsefu (−6.847791, −79.818263)

*Note:* DMS = geographic coordinate system degrees, minutes, seconds.

### 2.3. Laboratory Animals

*A. aegypti* larvae were collected from water reservoirs where mosquito breeding sites were found in the Olmos District of the department of Lambayeque. They were then taken to the Biotherium of the Hospital Regional Lambayeque for recognition and rearing under adequate environmental conditions of temperature (28°–30°), humidity (70%), 12 h of light, 12 h of darkness, and food (fish protein) in quantities according to the larval stage [9, 10]. A sample of specimens was sent to the Museo de Historia Natural VBA of the Universidad Nacional Pedro Ruiz Gallo for identification and recording. The female insects used in the bioassays were from the second to the fifth generation.

The specimens of *Rattus norvegicus albinus* were also reared in the Biotherium of the same hospital in an environment with standard conditions of temperature, light/darkness, water, and food *ad libitum*. When the male specimens met the established weight criteria (200–250 g) to be used as bait, they were randomly selected and exposed only once to the laboratory bioassay. The experiments were carried out observing the recommendations of the guide established by the Instituto Nacional de Salud del Peru [11].

### 2.4. Formulation of Essential Oils

The formulation of the essential oils was intended to solubilize them in water and facilitate the process of impregnation into the mosquito nets. Liquid samples of 100 mL were used: *O. vulgare* (Batch FMO20P8020), *E. globulus* (Batch FMEC20T207), *M. piperita* (Batch FMM20T206), and *C. citratus* (Batch FHL1024). All essential oils were formulated in a 1:1 ratio, using an ethyl alcohol solution as the main solvent and 8% polysorbate as an emulsifier to homogeneously disperse the compounds in aqueous media and promote their stability during the laboratory bioassay.

### 2.5. Impregnation of Mosquito Nets

White polyester mosquito netting with 60 holes per square centimeter was conditioned in 25 cm × 35 cm pieces (tesa). With the aim of softening and removing impurities, the pieces were prepared by washing and scrubbing with distilled water, then dried in a 35°C incubator for 90 min, and placed in double zipper Ziploc‐type bags. Finally, they were stored at room temperature and relative humidity of 70%–80%, until their use in the impregnation of the formulations. The impregnation procedure was carried out by the absorption method, following the recommendations described by Rastogi et al. [12], with some modifications:

50 mL of each formulation at concentrations of 250, 100, and 10 mg/mL was prepared in amber glass bottles with screw cap using distilled water as diluent. Two previously prepared mesh pieces were then introduced in small bundles and shaken vigorously for 10 min, keeping the bottle tightly closed. Once the bundles were removed from the bottle, they were pressed and the excess was removed using a glass roller on a 15 × 40 cm stainless steel tray. Then, the pieces were dried in the incubator at 35°C for 90 min, avoiding cross‐contamination between formulations. Finally, the twice‐folded pieces were stored and labeled in double zipper Ziploc‐type bags. A similar procedure was performed for the impregnation of the positive control (100 mg/mL DEET) and for the negative control with the diluent. All Ziploc bags containing the impregnated nets were stored in a cool, dry place until their use in the laboratory bioassay 24 h later.

### 2.6. Evaluation of Repellent Effect

For the repellency test, two‐compartment cages measuring 25 cm × 25 cm x 40 cm were used, separated by two removable acrylic frames. One of these frames had nine 20‐mm diameter holes, equidistant from each other, according to previously described specifications [5]. This cage was an adaptation of the one described by the WHO for evaluating the effectiveness of treated nets using tunnel testing [13]. One compartment contained 60 adult female *A. aegypti* mosquitoes, 5–8 days old and 12 h of starvation. The other compartment contained the *Rattus norvegicus albinus* bait sedated with 0.250 mL of intramuscular 6.5% sodium pentobarbital (Halatal), which was covered with a mosquito net impregnated with an essential oil formulation or control. The distribution of the cages for each treatment was randomized and blinded for the observers.

At the start of the test, the unperforated acrylic spacer frame was removed, thus enabling the mosquitoes to fly freely and through the holes in the second frame to reach the bait in search of food (blood). Successful landings of the insect to the mosquito net–covered bait were observed for 5 minutes and counted. After the five‐minute lapse, the acrylic frame was reassembled to its place, waiting for the next interval reading to repeat the procedure. The readings were taken every 30 min on the same mosquitoes, starting at 8:00 a.m. and ending at 11:00 a.m. Those that fed on the bait, showing a red and enlarged abdomen, were excluded from subsequent readings. The bioassays were performed under the following environmental conditions of 28°C–30°C temperature and 70% relative humidity.

The percentage of repellent effect was calculated using the following formula.

**FIGURE 2:**
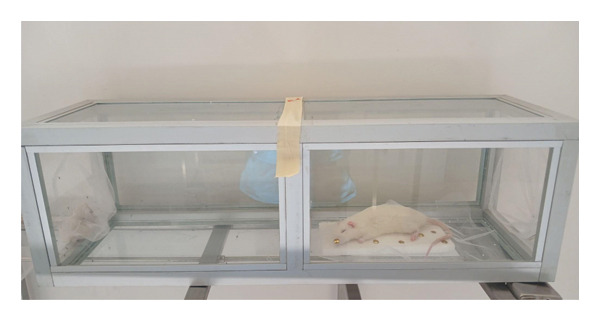
Two‐compartment cage repellency test configuration.

Repellency (%) = (N‐R/N) × 100, where N is equal to cumulative number of mosquitoes landed on the negative control bait and R is the number of mosquitoes landed on that of the formulations in question or 100 mg/mL DEET (see Figure 2).

### 2.7. Evaluation of the Protection Time

The protection time was evaluated simultaneously with that of the repellent effect and in the same test setup. The protection time was measured as the interval from the start of the test until the first successful bite, or the second successful landing of the insect on the coated bait, even if it did not bite and feed. This time was also counted for five minutes, at 30‐minute intervals, starting at 8:00 a.m. and ending at 11:00 a.m.

### 2.8. Evaluation of Insecticidal Effect

The insecticidal effect was evaluated by accounting for the female adults of *A. aegypti* knocked down or died 24 h after 3 hours of exposure to a rodent bait covered with a mosquito net impregnated with a formulation or control. For this purpose, the same test setup for the repellent effect was considered for exposure. At the end of the exposure period, the bait was removed from the cage, and 15% sucrose solution was used for *ad libitum* feeding until reading the following day. The insecticidal effect was measured as a percentage considering the proportion of knocked down or dead insects relative to the 60 used in the test. This percentage was adjusted with those counted in negative control, which should not exceed 10% of the total to consider the run valid. The surviving insects were removed from the test cages and placed in another cage where they were euthanized with 10% formaldehyde.

### 2.9. Statistical Analysis

Data were recorded in a Microsoft Office Excel 2019 spreadsheet. After assessing the normal distribution of the data, nonparametric estimates were made for both descriptive and inferential analyses. Descriptive statistics were generated by calculating the median for the repellent effect, protection time, and insecticidal effect for each experimental group and concentration tested. Inferential statistics were also calculated to compare the repellent effect, protection time, and insecticidal effect between groups using the Kruskal–Wallis nonparametric analysis of variance and Dunn’s test for multiple comparisons. A confidence level of 95% and statistical significance level of *p* < 0.050 were considered. The statistical software InfoStat/E 2020 version was used for the analysis.

### 2.10. Ethical Considerations

The protocol was reviewed and approved by the Institutional Ethics Committee for the Use of Animals (CEIPUA, for its Spanish initials) of the Hospital Regional Lambayeque with certificate no. 1‐2024. It was declared to know the ethical principles of research and the standards for animal care, which were observed throughout the research process. The rodents that participated in the laboratory bioassay were euthanized with 1 mL of intramuscular 6.5% sodium pentobarbital (Halatal).

## 3. Results

**FIGURE 3:**
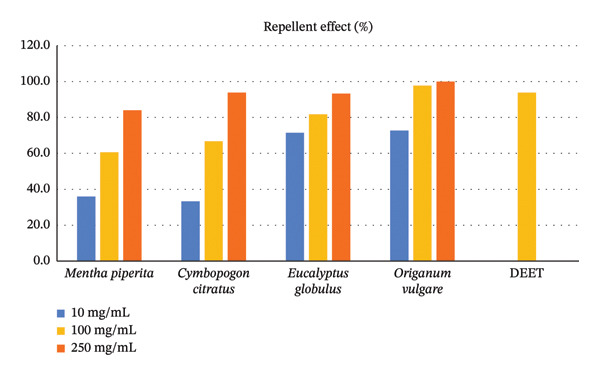
Repellent effect of formulations of essential oils and DEET impregnated into mosquito nets against the adult stage of *Aedes aegypti*.

**FIGURE 4:**
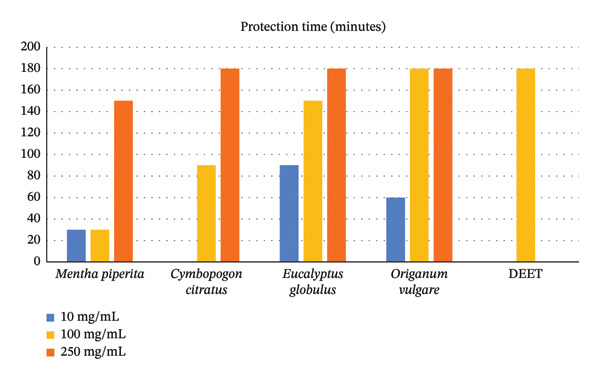
Protection time of formulations of essential oils and DEET impregnated into mosquito nets against the adult stage of *Aedes aegypti*.

**FIGURE 5:**
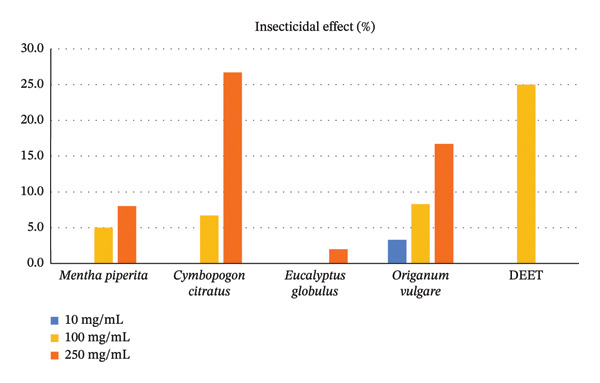
Insecticidal effect of formulations of essential oils and DEET impregnated into mosquito nets against the adult stage of *Aedes aegypti*.

The repellent and insecticidal efficacy of water‐soluble formulations of essential oils was determined through triplicate laboratory bioassays. The highest repellent effect was seen on *C. citratus*, *E. globulus,* and *O. vulgare* at a concentration of 250 mg/mL (93.3%–100.0%) (see Table 3 and Figure 3). Furthermore, the protection time was similar, where these three formulations achieved 180 min at the highest concentration tested (see Table 3 and Figure 4). Regarding the insecticidal effect, at a concentration of 250 mg/mL, *C. citratus* showed the greatest effect (26.7%), followed by *O. vulgare* (16.7%). For its part, DEET at 100 mg/mL achieved 93.3% repellency, 180 min of protection, and 25% insecticidal effect (see Table 3 and Figure 5).

**TABLE 3:** Repellent effect, protection time, and insecticidal effect of formulations of essential oils and DEET impregnated into mosquito nets against the adult stage of *Aedes aegypti*.

Group		Repellent effect (%)			Protection time (min)			Insecticidal effect (%)		
		10 mg/mL	100 mg/mL	250 mg/mL	10 mg/mL	100 mg/mL	250 mg/mL	10 mg/mL	100 mg/mL	250 mg/mL
*Mentha piperita*	Median	36.0	60.6	84.0	30	30	150	0.0	5.0	8.0
	Q1–Q3	23.8–54.5	42.9–76.0	71.4–84.8	30–30	30–30	120–180	0.0–0.0	1.7–6.0	1.7–8.3
*Cymbopogon citratus*	Median	33.3	66.7	93.9	0	90	180	0.0	6.7	26.7
	Q1–Q3	20.6–51.1	61.8–81.8	91.2–97.8	0–30	60–90	150–180	0.0–5.0	2.0–10.0	14.0–30.0
*Eucalyptus globulus*	Median	71.4	81.8	93.3	90	150	180	0.0	0.0	2.0
	Q1–Q3	57.8–75.8	76.2–86.7	90.9–100.0	60–90	120–150	150–180	0.0–0.0	0.0–0.0	0.0–5.0
*Origanum vulgare*	Median	72.7	97.7	100.0	60	180	180	3.3	8.3	16.7
	Q1–Q3	45.0–75.8	93.3–97.0	97.0–100.0	60–60	180–180	180–180	1.7–5.0	6.7–10.0	15.0–23.3
DEET	Median	NA	93.9	NA	NA	180	NA	NA	25.0	NA
	Q1–Q3	NA	93.3–97.0	NA	NA	150–180	NA	NA	22.0–33.3	NA

*Note:* Triplicate laboratory bioassay using 60 *Aedes aegypti* specimens per experimental group.

Abbreviations: DEET, N,N‐diethyl‐meta‐toluamide; min, minutes; NA , data not available; Q1–Q3, quartile 1–quartile 3.

Regarding the comparison of the repellent effect, the highest effect was seen in the *O. vulgare* formulation and DEET, with percentages higher than 94%, which had a higher effect than the *M. piperita* formulation (60.6%) (*p* = 0.019). The *C. citratus* and *E. globulus* formulations had an intermediate repellent effect, with 66.7% and 81.8%, respectively. Furthermore, the repellent effect was directly proportional to the concentration of the formulation used, being significantly different from each other (*p* < 0.001). Findings similar to the repellent effect were seen in the protection time, for which the longest time was seen in the *O. vulgare* formulation and DEET, both with 180 min, a time greater than that observed in the *M. piperita* formulation (30 min) (*p* = 0.030). The *C. citratus* and *E. globulus* formulations had an intermediate protection time, with 90 and 150 min, respectively. Likewise, the protection time was directly proportional to the concentration tested and different from each other (*p* < 0.001). Regarding the insecticidal effect, considering the three tested concentrations of the essential oil formulations, none achieved the mortality rate of DEET. The *E. globulus* and *M. piperita* formulations had a lower effect than *C. citratus* and *O. vulgare*, and these, in turn, were lower than the effect of DEET (*p* < 0.001) (see Table 4).

**TABLE 4:** Repellent effect, protection time, and insecticidal effect of formulations of essential oils impregnated into mosquito nets against the adult stage of *Aedes aegypti*.

Variables	*n*	Repellent effect (%)			Protection time (minutes)			Insecticidal effect (%)		
		Median	Ranges	*p* value	Median	Ranges	*p* value	Median	Ranges	*p* value
*Group*										
*Mentha piperita*	9	60.6	11.7 a	0.019	30	12.4 a	0.030	0	9.2 a	< 0.001
*Cymbopogon citratus*	9	66.7	16.3 ab		90	15.9 ab		1.7	16.1 a	
*Eucalyptus globulus*	9	81.8	21.4 ab		150	22.3 ab		6.7	23.3 b	
*Origanum vulgare*	9	97.0	27.1 b		180	26.0 b		8.3	26.0 b	
DEET	3	93.9	30.3 b		180	29.8 b		25.0	36.3 c	

*Concentration (mg/mL)*∗										
10	12	52.8	8.5 a	< 0.001	45	9.0 a	< 0.001	0	10.4 a	< 0.001
100	12	79.0	19.8 b		105.0	19.0 b		5.5	19.1 a	
255	12	93.6	29.2 c		180.0	29.5 c		11.2	26.4 b	

*Note:* The letters a, b, and c indicate significantly different ranges (Dunn’s test, *p* < 0.05); the *p* value corresponds to the Kruskal–Wallis test; DEET = N,N‐diethyl‐meta‐toluamide.

^∗^It includes only the formulations of essential oils.

## 4. Discussion

This study observed that the repellency rates and the protection time of water‐soluble formulations of essential oils impregnated into mosquito nets were equivalent to 100 mg/mL DEET, depending on the concentration used, achieving efficacies of 84%–100% and 180 min at its highest concentration tested (250 mg/mL). These effects could be explained by the action of one or more of its aromatic compounds, namely, coumarins, tannins, anthraquinones, and saponins in *C. citratus* [14], 1,8‐cineole in *E. globulus* [15], menthol, menthone, neomenthol, and isomenthone in *M. piperita* [16], and thymol, terpinene, and carvacrol, among others, in *O. vulgare* [17]. However, the mechanisms of action in the insect are still unclear [18].

In this regard, previous studies have reported the repellent and larvicidal effect of the essential oils from *C. citratus* [14] and *M. piperita* [19] against *A. aegypti*. Their effectiveness depends on their concentration and environmental conditions. Also, other studies have revealed the repellent potential of the essential oils from *E. globulus* [5, 8, 20], *C. citratus* [8, 21, 22], and *M. piperita* [8] against *A. aegypti* adults, for which rates between 50% and 100% were reported. Regarding the essential oil from *O. vulgare*, no study has been found that evaluates its repellent effect on *A. aegypti* adults, but its repellent activity against other insects such as *Cimex lectularius* [17], *Anobium punctatum* [23], and *Tribolium confusum* [24] has been reported.

On the other hand, 100 mg/mL DEET impregnated into mosquito nets showed a repellency higher than 90%. This synthetic compound is considered the reference repellent, and although the pathophysiological mechanisms of its action are not yet clear, it is probable that more than one is involved, among which are sensory (olfactory or contact) and physiological disturbances that affect the normal reception of vertebrate kairomones, altering central nervous transmission [18].

Regarding protection time, it was observed that it increased with concentration, reaching 180 min at the highest concentration tested in the oils of *O. vulgare*, *C. citratus*, and *E. globulus*, like the time observed with 100 mg/mL DEET. While no studies have been found on impregnated water‐soluble formulations, there are reports that evaluate essential oils in their natural state. Thus, a study conducted in Peru reported that the essential oils of *C. citratus*, *E. globulus*, and *M. piperita* showed a protection time exceeding 100 min at a concentration of 250 mg/mL and similar efficacy to that obtained with 100 mg/mL DEET [8]. Another study in Colombia reported that *C. citratus* at 40% had a maximum protection time of 90 min [20], while another report in the same country reported a protection time of 120 min at 0.6% [19]. On the other hand, in Madagascar, 100 mg/mL DEET was found to repel more than 80% of mosquitoes for up to 120 min [25]. In addition, it has also been reported that 250 mg/mL DEET has a longer protection time than eucalyptus essential oil at the same percentage [26]. The differences found are due to the type of exposure protocol, the origin of the active compounds, and the *A. aegypti* strains used.

These results suggest that, at appropriate concentrations, the tested formulations are effective repellents comparable to DEET, provided they are reapplied according to the observed protection intervals [27, 28]. Furthermore, this study proved that the water‐soluble formulation of the essential oils was successfully impregnated into the mosquito net, and that this procedure did not significantly affect its repellent activity. Therefore, this type of formulation has the advantage of being usable in the field for topical applications or for impregnation into fabrics by absorption methods. However, it will be necessary to evaluate the volatility rate by comparing it with other types of formulations [28].

Regarding the insecticidal effect on *A. aegypti* adults, none of the formulations tested showed significant efficacy, nor did DEET. This finding could be explained by the type of exposure to which the insects were subjected, i.e., indirect contact with the volatile compounds or by landing on the impregnated net covering the bait when they tried to feed. Notwithstanding these findings, several previous studies have reported the insecticidal activity of these essential oils in their natural state against other insects and through different methodologies [24, 29].

According to the evidence, the effect of the formulations can vary depending on several factors, mainly the active compounds, the concentration and type of formulation of the essential oil, the species of mosquito, the type of exposure, and the test setting [30]. On the other hand, a recent study in terrestrial isopods showed lower acute toxicity of these essential oils compared to DEET and cypermethrin, suggesting environmentally friendly advantages in essential oils [31].

The reduced number of insects per test (60 *A. aegypti* females) and the minimal number of replicates (triplicate) was a limitation of the study due to possible random errors introduced, but they are usual in this type of designs for ethical and logistical reasons. Likewise, due to logistical reasons, the phytochemical components of the essential oils used for formulations were not characterized. Although the components of these essential oils have already been described, the types and quantities vary according to the geographical origin of the plants. However, we consider the results to be valid, reproducible, and plausible. Furthermore, the use of rats as bait for an anthropophilic insect like *A. aegypti* may have introduced bias into the repellency measurement; however, the inclusion of controls under the same experimental conditions mitigated this bias. This laboratory bioassay provides evidence of the repellent effect of water‐soluble formulations of essential oils, but it is necessary to test them in humans, as well as their acceptability and adverse effects.

It is concluded that the water‐soluble formulations of essential oils from *C. citratus*, *E. globulus,* and *O. vulgare* at 250 mg/mL and 100 mg/mL DEET impregnated into mosquito nets showed a similar repellent effect and protection time against *Aedes aegypti* female adults. However, none showed a significant insecticidal effect on the same insect. The similar repellency of *C. citratus*, *E. globulus*, and *O. vulgare* formulations to DEET supports their potential as alternatives for mosquito control. Further studies should assess their stability, safety, and acceptability under field conditions.

## Author Contributions

All authors contributed equally to the conception and collection of samples and data for the study. H.S.D. performed the analysis and interpretation of the data. All authors drafted the manuscript and participated in its critical review. Likewise, all authors are responsible for the results reported.

## Funding

This study was supported by Universidad de San Martín de Porres, approved project code E2110202309.

## Disclosure

All authors approved this final version of the report.

## Conflicts of Interest

The authors declare no conflicts of interest.

## Data Availability

The data that support the findings of this study are available from the corresponding author upon reasonable request.
